# Computational Study of the Ir-Catalyzed Formation of Allyl Carbamates from CO_2_

**DOI:** 10.1021/acs.organomet.4c00177

**Published:** 2024-08-05

**Authors:** Sahil Gahlawat, Markus Artelsmair, Abril C. Castro, Per-Ola Norrby, Kathrin H. Hopmann

**Affiliations:** † Department of Chemistry, UiT The Arctic University of Norway, N-9017 Tromsø, Norway; ‡ Hylleraas Centre for Quantum Molecular Sciences, UiT The Arctic University of Norway, N-9017 Tromsø, Norway; § Isotope Chemistry, Early Chemical Development, Pharmaceutical Sciences, R&D, AstraZeneca Gothenburg, SE-431 83 Mölndal, Sweden; ∥ Department of Chemistry and Hylleraas Centre for Quantum Molecular Sciences, University of Oslo, P.O. Box 1033 Blindern, 0315 Oslo, Norway; ⊥ Data Science and Modelling, Pharmaceutical Sciences, R&D, AstraZeneca Gothenburg, SE-431 83 Mölndal, Sweden

## Abstract

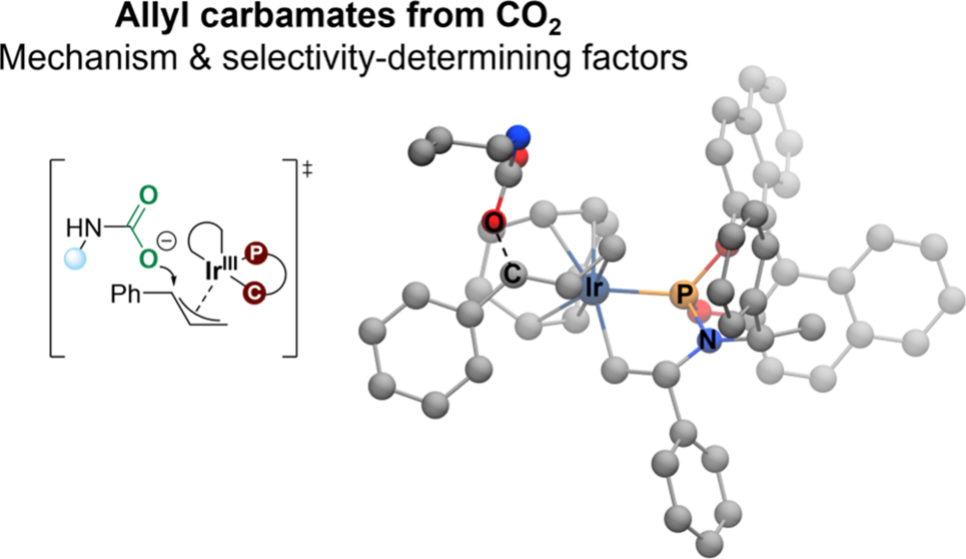

We have employed computational methods to investigate the iridium-catalyzed allylic substitution leading to the formation of enantioenriched allyl carbamates from carbon dioxide (CO_2_). The reaction occurs in several steps, with initial formation of an iridium-allyl, followed by nucleophilic attack by the carbamate formed in situ from CO_2_ and an amine. A detailed isomeric analysis shows that the rate-determining step differs for the (*R*)- and (*S*)-pathways. These insights are essential for understanding reactions involving enantioselective formation of allyl carbamates from CO_2_.

## Introduction

Carbon dioxide (CO_2_) has become a valuable C1 synthon for the synthesis of organic molecules. Conventional carbon sources such as crude oil and coal are finite and nonrenewable, whereas CO_2_ is abundant and nontoxic.^1^ Around 35 billion tonnes of CO_2_ were emitted in 2020 due to the burning of fossil fuels.^2^ The International Energy Agency outlines that in order to achieve net zero emissions by 2050, carbon capture, utilization, and storage (CCUS) will play a critical role.^3^ These points provide relevant reasons to produce materials of commercial interest from CO_2_.

Despite the advances made in the use of CO_2_ in synthetic organic chemistry, it is still a challenge to design enantioselective reactions with CO_2_ to form chiral products.^4^ Chiral molecules in their enantioenriched form are extensively used in pharmaceutical industries,^5,6^ and they have ubiquitous applications, ranging from medicinal chemistry to material science.^7^ However, asymmetric synthesis is difficult, and the infinitesimal steric effects of CO_2_ make it hard to capture it in an enantioselective manner.^8,9^ Some catalytic enantioselective reactions involving CO_2_ as a C1 synthon have been reported, leading to the formation of chiral carboxylic acids,^10^ esters,^100^ carbonates,^11,12^ and carbamates.^13^

**Scheme 1:**
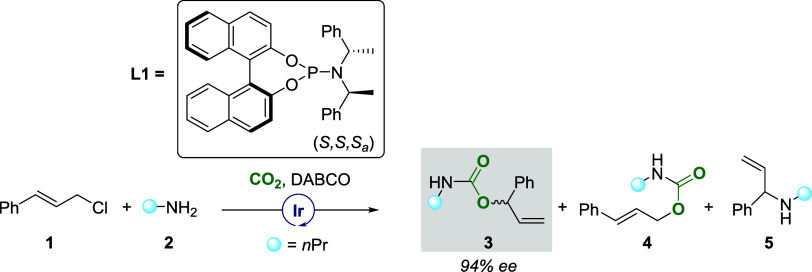
Previously Reported Asymmetric Allylic Substitution Reaction Involving an Iridium-Based Catalyst Coordinated by a Chiral Phosphoramidite Ligand L1^18^ DABCO = 1,4-diazabicyclo[2.2.2]octane, 1 (0.24 mmol, 120 mol %), 2 (0.20 mmol, 100 mol %), ratio of products 3:4 = 90:10, yield of product 5 = 13%.

Organic carbamates are an important class of compounds often found in natural products, medicines, agricultural chemicals, and pharmaceuticals.^14−16^ A synthetic route for the enantioselective formation of cyclic carbamates from secondary amines and CO_2_ facilitated by an organocatalyst was reported by Yousefi et al.^17^ Liu et al. achieved the synthesis of acyclic carbamates with high enantiopurity from CO_2_ and *meso*-epoxides via polycarbonate intermediates aided by a dinuclear Co(III) complex.^19^ Zhao and co-workers produced branched allylic carbamates with high enantiopurity under mild conditions,^18^ using an asymmetric domino reaction of CO_2_ with allyl chlorides and primary amines catalyzed by an iridium complex featuring Feringa’s ligand (**L1**, Scheme 1).^20,21^

The allylic substitution reaction aided by an Ir-based catalyst to give branched enantioselective products has been well-known since the late 1990s.^22,23^ There have been experimental studies on the active catalyst species^24,25^ and the origin of the enantioselectivity in reactions between different allylic substrates (acetates, benzoates, carbonates) and amine nucleophiles.^26^ A computationally driven study of the iridium-catalyzed allylic substitution showed that the regioselective formation of branched products results from noncovalent interactions between the allyl ligand and the incoming nucleophile.^27^ However, a complete mechanistic study is lacking, especially with CO_2_ as a cosubstrate. In particular, for the reaction reported by Zhao and co-workers (Scheme 1),^18^ it is relevant to elucidate how the allylic chloride is activated by the iridium complex, how amine and CO_2_ combine to form the nucleophilic carbamate species, as well as the details of the enantioselective insertion of the latter into the iridium-allyl bond. A mechanistic study of this reaction is challenging, as there are many starting reagents, including allyl chloride, primary amine, CO_2_, and base, which implies that there are various possible reaction routes. It is also noteworthy that two experimental studies employing the same (*S*,*S*,*S*_a_)-**L1**-iridium catalyst have reported different enantioselectivities in the reaction of cinnamyl chloride, propylamine, and CO_2_, with the enantiomeric excess (*e.e.*) of the resulting allyl carbamate reported as 94% (*R*) and 35% (*S*), respectively.^18,28^

Here, we have conducted a detailed computational study of the enantioselective iridium-catalyzed formation of allylic carbamates from CO_2_ using state-of-the-art density functional theory (DFT) methods. The stereoselectivity and regioselectivity of the reaction were thoroughly analyzed to understand the underlying mechanism and deduce the major product. A detailed isomeric study of the transition states in the computed mechanism shows that the rate-determining step differs for the diastereomeric pathways, leading to the formation of the product enantiomers. Our results also provide an understanding of the kinetic competence of different possible intermediates in the allylic substitution reaction catalyzed by iridium-(phosphoramidite) complexes.

## Computational Details

**Figure 1:**
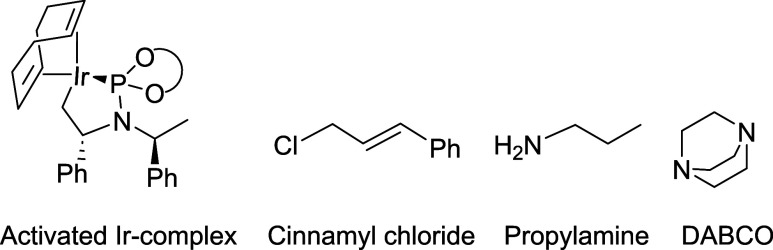
Computational model employed: the Ir-complex with the activated ligand **L1**, the cinnamyl chloride and propylamine substrates, and the base DABCO.

Calculations were performed on complete molecular systems without any truncations (Figure 1). The software used for all DFT calculations was Gaussian16 (Revision B.01).^29^ The systems were fully relaxed, and no symmetry constraints were imposed. For the geometry optimizations, the hybrid PBE0 functional^30,31^ containing 25% Hartree–Fock exchange was used, along with Grimme’s D3(BJ) dispersion correction.^32^ The BS1 basis set was used for geometry optimizations, which comprises the SDD basis set and effective core potential^33,34^ for iridium and def2-SVP^34−37^ for other elements. Frequency calculations confirmed the minimum and transition state (TS) structures. To refine the electronic energies, single-point calculations were performed using the BS2 basis set consisting of the SDD basis set and effective core potential for iridium and def2-TZVPP^34−37^ for other elements. Solvation effects were included in both geometry optimizations and single-point calculations using the polarizable continuum model (IEFPCM)^38^ with the parameters of toluene (ε = 2.37). The results obtained at the PBE0-D3(BJ) level of theory were compared to other DFT functionals, B3LYP-D3(BJ)^39^ and ωB97XD^40^ (see the Supporting Information for details). In addition, we performed ab initio molecular dynamics (AIMD) simulations of intermediate **B**_***re***_ to investigate the behavior of the released counterion (Cl^–^, see the SI for details). Reported Gibbs free energies (standard state, 1 atm) include thermal corrections computed at 298 K, which is considered a reasonable approximation to the experimental temperature of 288 K. The enantiomeric excess and regioselective ratios were calculated on the basis of the computed Gibbs free barriers of the C–O bond formation transition states, employing the Eyring equation.^41^ We visualized the noncovalent interactions between carbamate and the iridium complex in relevant transition states with the NCIPLOT 4.0 program.^42^ The density and gradient files generated by the program were used to draw the isosurface displaying the interactions.

## Results and Discussion

### Allylic Substitution Mechanism

**Scheme 2:**
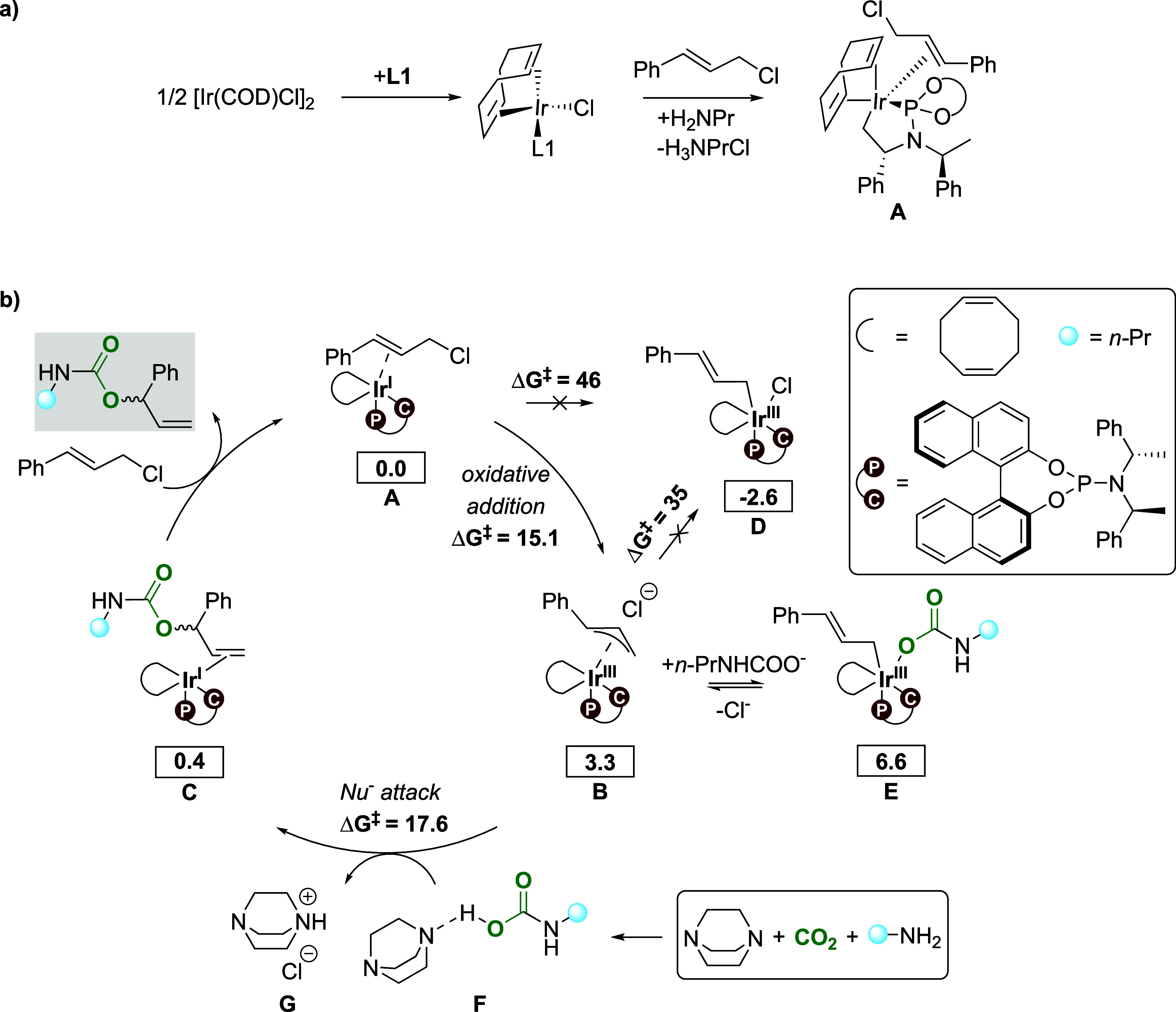
(a) Previously Proposed Active Catalyst Species A.^24,43^ (b) Proposed Reaction Cycle for the Ir-Catalyzed Allylic Substitution to Furnish Allyl Carbamates Using CO_2_ Based on Our Computed Results and Reported Mechanisms^28,44^ Free energies computed at 298 K (values in kcal/mol, PBE0-D3(BJ)/def2-TZVPP,SDD[Ir](PCM)//PBE0-D3(BJ)/def2-SVP,SDD[Ir](PCM) level of theory). The energetic reference state for the mechanistic cycle is complex **A** plus adduct **F**.

We have computationally studied the enantioselective and regioselective iridium-(phosphoramidite)-catalyzed conversion of CO_2_, propylamine, and cinnamyl chloride to allyl carbamates (Scheme 1), with a plausible mechanism proposed in Scheme 2. Initially, an active species is formed, as outlined in Scheme 2a. The mixture of [Ir(COD)Cl]_2_, phosphoramidite ligand (**L1**), and propylamine in THF solution leads to the formation of a cyclometalated iridium(I) species,^18,24^ which is assumed to be the active catalyst. The association of cinnamyl chloride **1** to the cyclometalated complex via a π-interaction gives the η^2^ iridium(I) complex **A**. The formation of **A** is in line with other studies proposing a similar active species.^25,43^ The allylic substitution reaction can then proceed, as proposed in Scheme 2b, based on our computational results and suggestions from related mechanisms in the literature.^28,44^ An alternative pathway involving the initial coordination of the amine to the iridium complex was also evaluated but was found to be nonfeasible (see the SI, Scheme S1).

**Figure 2:**
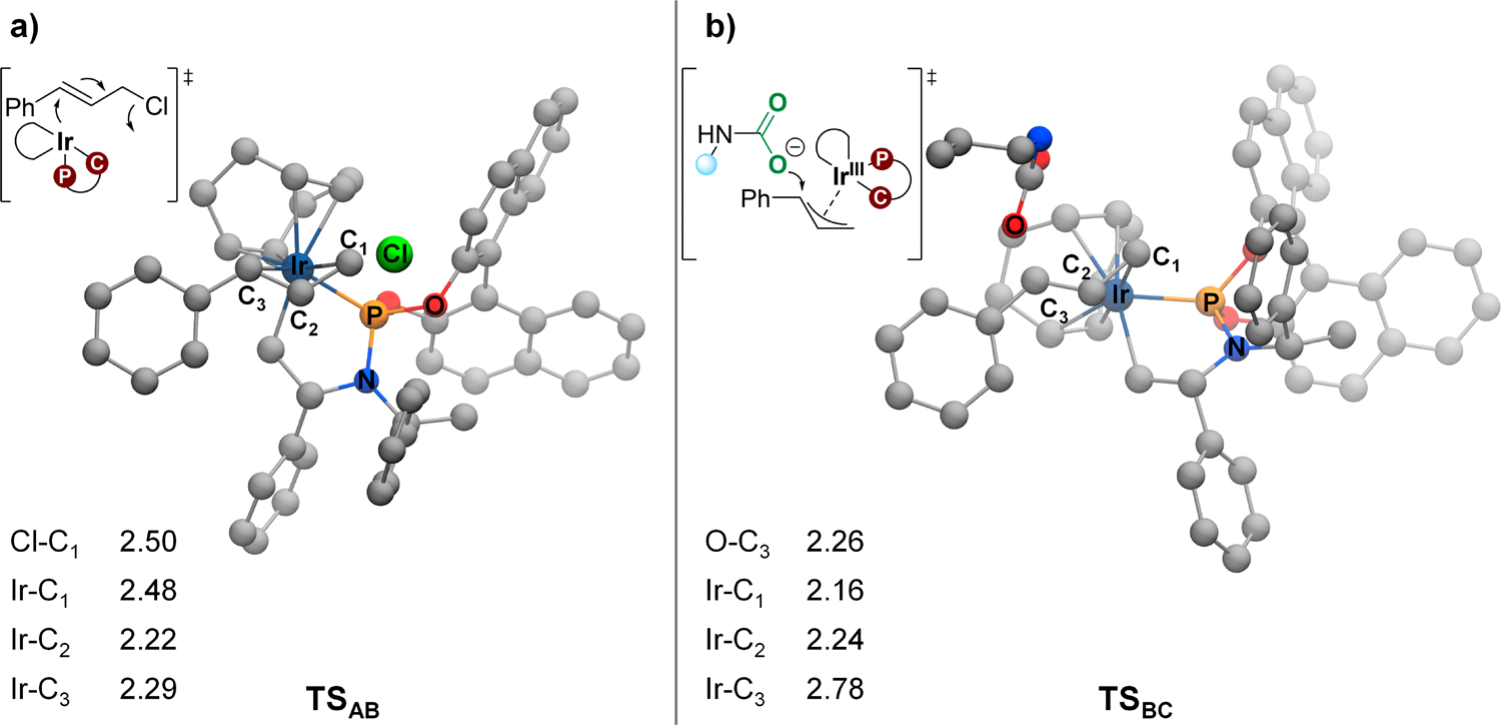
Optimized transition state geometries for (a) oxidative addition of cinnamyl chloride and (b) nucleophilic attack of carbamate on the allyl for the (COD)(**L1**)-Ir-catalyzed allylic substitution of **1** (distances are given in Å).

From cinnamyl chloride-coordinated complex **A**, the proposed catalytic cycle starts with a formal oxidative addition of cinnamyl chloride to the Ir(I) complex (Scheme 2b and Figure 2a). Interestingly, in our calculations, this step proceeds through an S_N_2-type oxidative addition mechanism,^45^ furnishing an (η^3^-allyl)iridium(III) complex **B** and free chloride, with a barrier of only 15.1 kcal/mol (Scheme 2b). An alternative pathway proceeding through a concerted oxidative addition of cinnamyl chloride to **A** was found to be not feasible (barrier of Δ*G*^‡^ = 46 kcal/mol, Scheme 2b). In the formed intermediate **B**, the liberated Cl^–^ ion makes weak interactions with the ligands. Additional ab initio molecular dynamics (AIMD) simulations support the notion that the Cl^–^ ion does not coordinate to iridium in **B** (see the SI for details). Subsequent coordination of chloride to iridium to give the η^1^-allyl intermediate **D** is thermodynamically favorable. However, the conversion of **B** to **D** has a barrier of 35 kcal/mol (31.7 kcal/mol relative to **B**). Although this barrier is in line with other reported η^3^ to η^1^ allyl conversions,^46^ it is too high to be feasible at the experimental temperature. We therefore propose that the potential chloride-coordinated intermediate **D** is not formed. This is in agreement with other mechanistic proposals suggesting that the leaving group of the allylic substrate does not coordinate to iridium.^24,44^

Simultaneously with the oxidative addition of the allyl chloride, we propose that the free CO_2_ and propylamine **2** in the reaction mixture combine to carbamic acid, with the aid of the base DABCO, to form the DABCO-propyl carbamic acid adduct **F**. This process is endergonic by 0.5 kcal/mol (see the SI for a comparison of energies of other species). We investigated whether the iridium complex may be involved in the formation of carbamic acid but concluded that this appears less likely than the direct reaction of CO_2_ and propylamine (for details, see the SI, Scheme S3). This is in line with other results, showing that CO_2_ and alkyl amines can combine in the absence of a metal catalyst.^47,48^

The deprotonation of the formed propyl carbamic acid by DABCO and the elimination of the DABCOH^+^/Cl^–^ ion pair **G** provides an ionic propyl carbamate (Scheme 2b). This can potentially compete with the η^3^-allyl in **B** to give the off-cycle η^1^-allyl species **E**, which is a slightly endergonic process. We could not locate a relevant TS for the formation of **E**. However, even if it is formed, it would have to convert back to **B** for the allylic substitution reaction to proceed. Therefore, we propose that the ionic propyl carbamate performs an S_N_2-like nucleophilic attack on the allyl fragment of **B** to provide iridium(I) intermediate **C**, featuring an allyl carbamate coordinated through the double bond (Scheme 2b). The nucleophilic attack can occur on different carbon atoms of the allyl, with the energetically preferred TS (Figure 2b) involving the attack at the benzylic position, with a barrier of 17.6 kcal/mol (relative to **A**, Scheme 2b).^49^ The subsequent displacement of the formed allylic carbamate **3** by cinnamyl chloride **1** concludes the catalytic cycle.

### Enantioselectivity in the Formation of **3**

**Figure 3:**
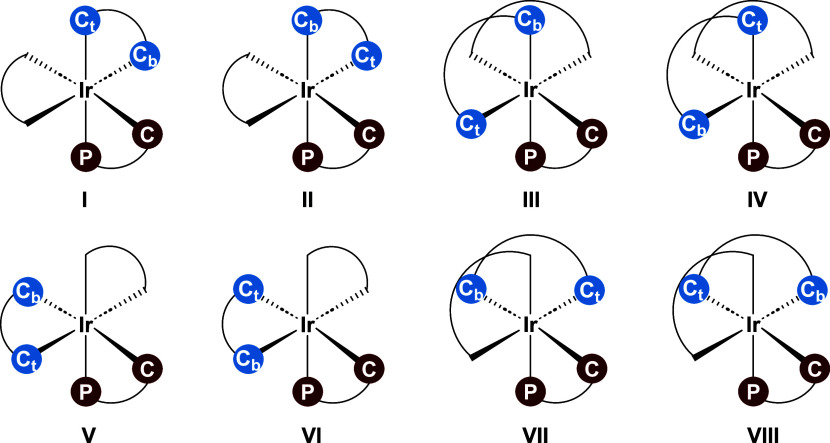
Eight possible isomers of **B**. The atom labels P and C (red) correspond to the phosphoramidite ligand; C_b_ and C_t_ (blue) represent the benzylic and terminal carbon atoms of the allyl, and the black curve denotes the COD ligand.

The selectivity of the overall allylic substitution process will either be determined through the formation of **B** (via the oxidative addition of cinnamyl chloride, **TS**_**AB**_) or through the conversion of **B** to **C** (via the nucleophilic attack on the allyl, **TS**_**BC**_), depending on the energies of the involved TSs.^50^ In order to gain insights into the selectivity-determining factors governing the formation of allyl carbamate product **3**, we conducted a systematic analysis of the isomers that can be formed from **TS**_**AB**_ and **TS**_**BC**_. The three iridium ligands (namely, COD, phosphoramidite, and allyl) in **B** bind in a bidentate fashion, forming a distorted octahedral geometry. Figure 3 illustrates the eight possible isomers (**I–VIII**) of **B** based on varying the position of the ligands around the iridium. We have thus analyzed **TS**_**AB**_ and **TS**_**BC**_ for all eight isomers (**I–VIII**), also taking into account the different binding modes of the substrate (*vide infra*).

**Figure 4:**
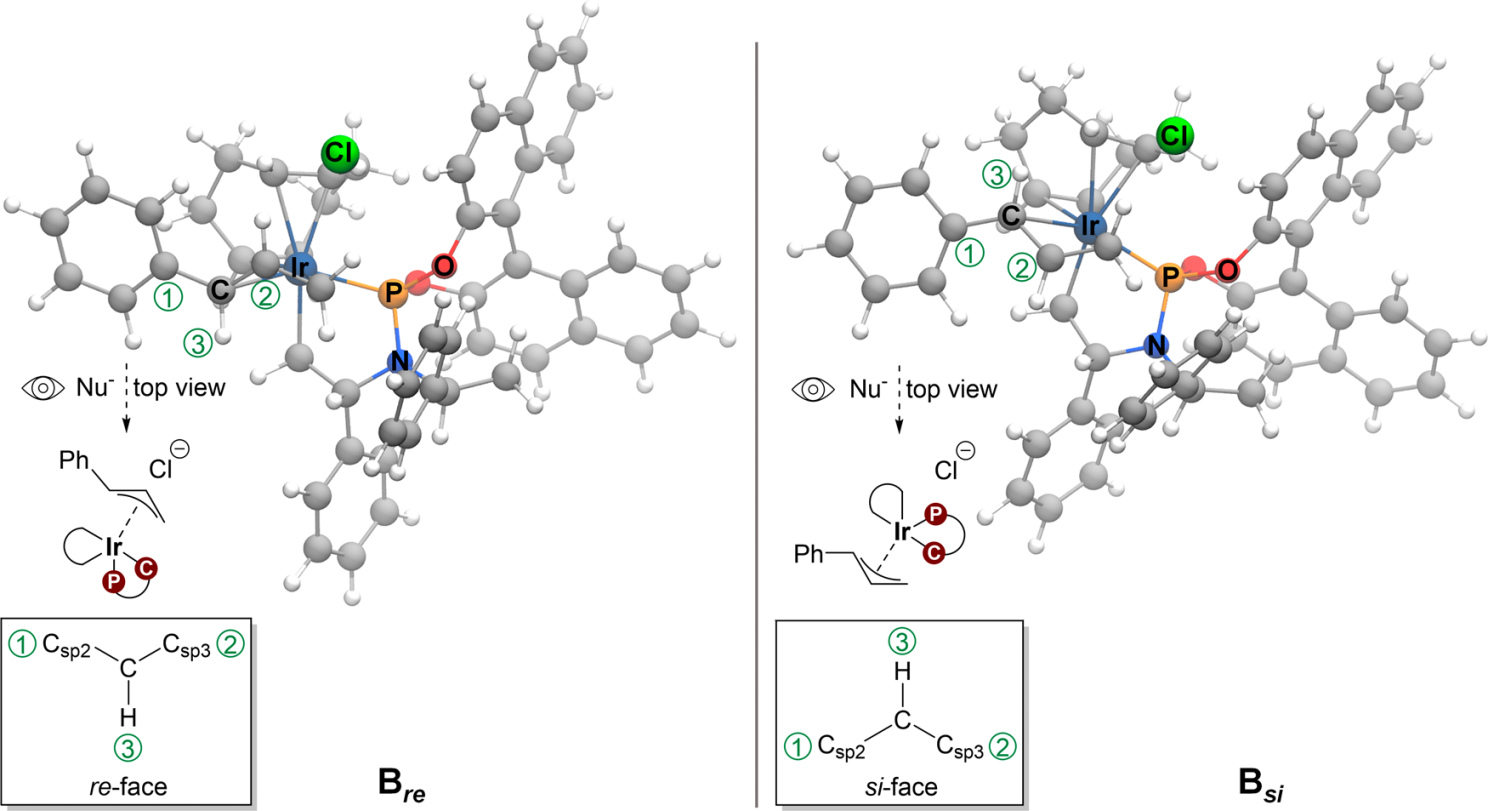
*re* and *si* allyl binding modes of complex **B** (isomer **II**). The binding mode with the *re* face accessible, as seen from the top view, is marked as the *re* mode. Similarly, the binding mode with the *si* face accessible is referred to as the *si* mode.

The cinnamyl chloride in starting complex **A** binds to iridium via a π-interaction to form a *re* or *si* face around the benzylic carbon, resulting in complexes **A**_***re***_ or **A**_***si***_. The subsequent S_N_2-like oxidative addition of cinnamyl chloride renders the (η^3^-allyl)iridium(III) complexes **B**_***re***_ or **B**_***si***_ (see Figure 4 for a visual representation). The *re* modes lead to the (*R*)*-*configuration of the final allyl carbamate product, whereas the *si* modes provide the (*S*)*-*isomer. The apparent rotation of the allyl to directly interconvert between binding modes is a known process for Pd-based complexes,^51^ but for Cp*Ir(CO)(C_3_H_5_) complexes, it has been shown that allyl interconversion is energetically inaccessible.^52^

Combining the eight isomers **I–VIII** (Figure 3) with the two possible allyl binding modes (*si* and *re*, Figure 4) results in a total of 16 transition states to take into account. Table 1 shows the computed **TS**_**AB**_ barriers for **I–VIII** relative to those of **A**_***si***_. Among the 16 isomers of **TS**_**AB**_, the computed energies show that the *si* mode of isomer **II** provides the lowest barrier (**TS**_**AB**_-**II**_***si***_ = 15.1 kcal/mol, Table 1), which is almost 5 kcal/mol lower than the next lowest barrier of 20.0 kcal/mol obtained for isomer **I** (**TS**_**AB**_-**I**_*si*_). Thus, we consider **II** as the most stable TS isomer for the oxidative addition of cinnamyl chloride to **A** (Figure 2a). We note that the benzylic carbon of the allyl fragment is *trans* to the phosphorus atom of the phosphoramidite ligand in isomer **II**. This finding differs from a previous experimental study, which suggested a favored *cis* binding mode of the allyl.^27^ We attribute the different results to variations in the ligand and allyl substrate employed in our study.

**Table 1:**
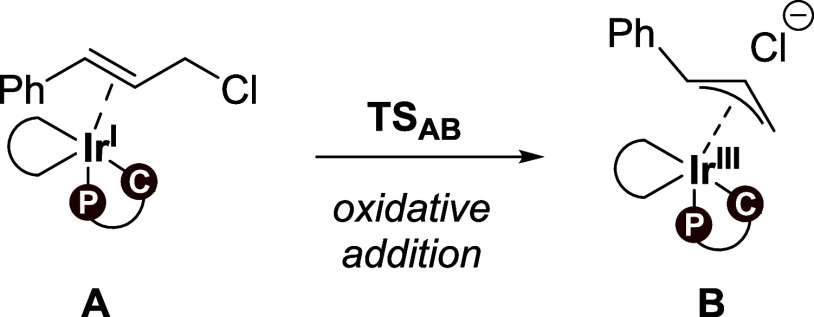
Calculated TS_AB_ Gibbs Free Energy Barriers (kcal/mol, Relative to A_*si*_) for the Addition of Cinnamyl Chloride to A Leading to Isomers I to VIII of B

TS_AB_	Δ*G*_*re*_^‡^	Δ*G*_*si*_^‡^	ΔΔ*G*^‡^^b^
**TS**_**AB**_**-I**	22.3	20.0	2.3
**TS**_**AB**_**-II**	20.3	15.1	5.2
**TS**_**AB**_**-III**	21.7	28.9	–7.2
**TS**_**AB**_**-IV**	22.6	24.7	–2.1
**TS**_**AB**_**-V**	27.5	22.3	5.2
**TS**_**AB**_**-VI**	20.1	n.d.^a^	-
**TS**_**AB**_**-VII**	33.0	28.8	4.2
**TS**_**AB**_**-VIII**	26.5	29.6	–3.1

a n.d. = TS could not be optimized.

b ΔΔ*G*^‡^ = Δ*G*_*re*_^‡^ – Δ*G*_*si*_^‡^

For the subsequent nucleophilic attack on **B**, we note that the geometric flexibility of the free propyl carbamate renders computation of the possible isomers of **TS**_**BC**_ challenging. Therefore, we analyzed **TS**_**BC**_ both with propyl carbamate (the experimental nucleophile) as well as with chloride as a model nucleophile, which does not have any inherent geometric flexibility. Importantly, the chloride ion attacks at the benzylic position of the allyl; hence, **TS**_**BC-Cl**_ (formation of a benzylic carbon-chloride bond) is not identical to **TS**_**AB**_ (cleavage of the terminal carbon-chloride bond). Table 2 displays the **TS**_**BC**_ barriers (relative to **A**_***si***_) leading to the (*R*)- and (*S*)-configurations of the products. Notably, the trend of both nucleophiles (propyl carbamate or chloride) is similar across the computed TS isomers, with isomer **II** providing the lowest barriers for the formation of both the allyl carbamate (17.5 kcal/mol for **TS**_**BC**_**-II**_**cb**_**_(__*R*_**_**)**_) and the allyl chloride (13.1 kcal/mol for **TS**_**BC**_**-II**_Cl_**_(_*****_S_*****_)_**). Interestingly, the two nucleophiles (propyl carbamate or chloride) appear to give markedly different enantioselectivities, with a clear preference for the (*S*)-product with chloride and an apparently racemic result for the formation of cinnamyl carbamate, as the energy difference between **TS**_**BC**_****-II**_**cb**_**_**(*****R*****)**_ and **TS**_**BC**_****-II**_**cb**_**_**(*****S*****)**_ is only 0.1 kcal/mol (Table 2). An analysis of the NCI plots for **TS**_**BC**_******-II**_**cb**_****_**(*****R*****)**_ and **TS**_**BC**_******-II**_**cb**_****_**(*****S*****)**_ shows comparable noncovalent interactions between the nucleophilic propyl carbamate and the iridium complex at the two TSs (SI, Figure S4), in line with the small energy difference. The apparent racemic result is in contrast to the experiment (*e.e.*s of 35 and 94%);^18,28^ however, we note that the enantioselectivities of the overall reaction are not determined only by **TS**_**BC**_, as discussed below.

**Table 2:**
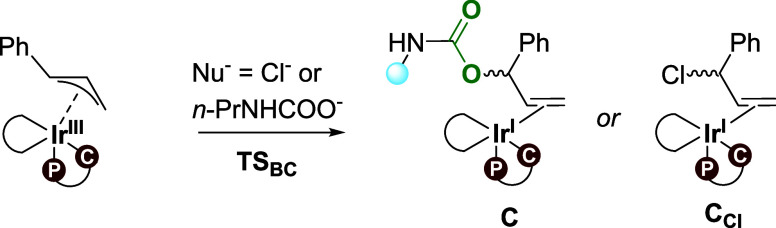
Calculated TS_BC_ Gibbs Free Energy Barriers (kcal/mol, Relative to *A*_si_) Leading to (*R*)- and (*S*)-Enantiomers of the Allyl Product^a^

TS_BC_	Δ*G*_*R*_^‡^	Δ*G*_*S*_^‡^	ΔΔ*G*^‡^^c^
**TS**_**BC**_**-I**_**cb**_	23.4	25.0	–1.6
**TS**_**BC**_**-II**_**cb**_	17.5	17.6	–0.1
**TS**_**BC**_**-III**_**cb**_	25.6	27.5	–1.9
**TS**_**BC**_**-IV**_**cb**_	27.6	30.1	–2.5
**TS**_**BC**_**-V**_**cb**_	24.9	23.1	1.8
**TS**_**BC**_**-VI**_**cb**_	23.8	n.d.^b^	–
**TS**_**BC**_**-VII**_**cb**_	28.7	29.6	–0.9
**TS**_**BC**_**-VIII**_**cb**_	32.0	27.5	4.5
**TS**_**BC**_**-I**_**Cl**_	20.2	23.6	–3.4
**TS**_**BC**_**-II**_**Cl**_	16.0	13.1	2.9
**TS**_**BC**_**-III**_**Cl**_	21.3	21.7	–0.4
**TS**_**BC**_**-IV**_**Cl**_	23.3	21.0	2.3
**TS**_**BC**_**-V**_**Cl**_	19.8	19.8	0.0
**TS**_**BC**_**-VI**_**Cl**_	20.4	22.4	–2.0
**TS**_**BC**_**-VII**_**Cl**_	26.0	22.9	3.1
**TS**_**BC**_**-VIII**_**Cl**_	24.0	25.2	–1.2

a Cl and cb subscripts represent the chloride and propyl carbamate nucleophiles, respectively.

b n.d. = TS could not be optimized.

c ΔΔ*G*^‡^ = Δ*G*_*R*_^‡^ – Δ*G*_*S*_^‡^

**Figure 5:**
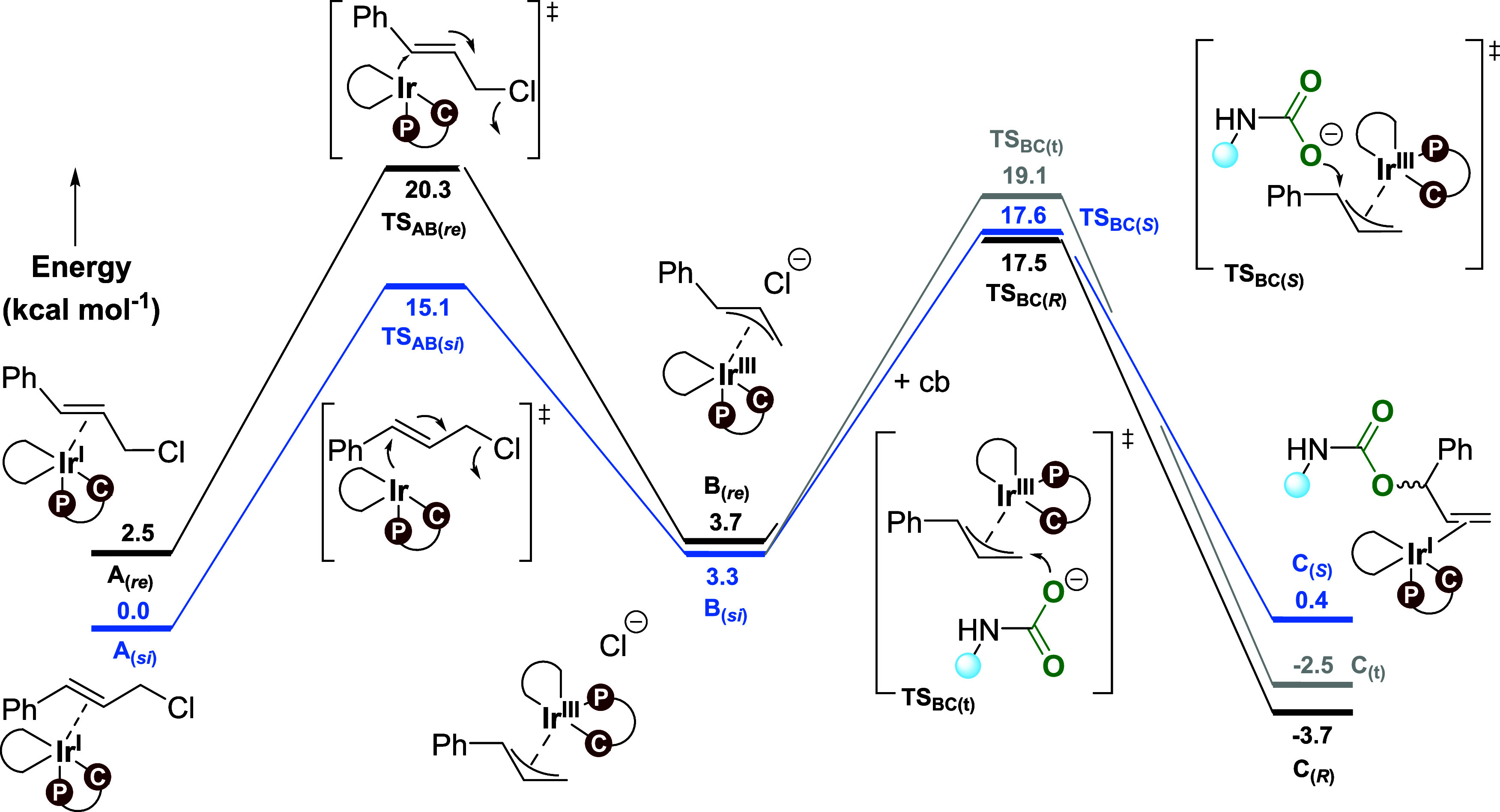
Gibbs free energy profile (based on isomer **II**) for the Ir-catalyzed allylic substitution to furnish allyl carbamates using CO_2_, including enantioselective and regioselective pathways (298 K), kcal/mol, PBE0-D3(BJ)/def2-TZVPP,SDD[Ir](PCM)//PBE0-D3(BJ)/def2-SVP,SDD[Ir](PCM). The profiles depicted in black and blue represent the formation of the (*R*)- and (*S*)-enantiomers of **3**, respectively, while the gray profile corresponds to **4**. The acronym cb denotes the carbamate nucleophile.

The energy profile for the full reaction with the preferred isomer **II** and propyl carbamate as the nucleophile is shown in Figure 5. **TS**_**AB(*****re*****)**_ and **TS**_**AB(*si*)**_ correspond to the oxidative addition TSs leading to a *re* or *si* face around the benzylic carbon in the iridium-π-allyl complex **B**, forming **B**_**(*****re*****)**_ and **B**_**(*****si*****)**_ complexes. **TS**_**BC(*****R*****)**_ and **TS**_**BC(*****S*****)**_ represent the TSs for the propyl carbamate attack on the allyl (isomer **II**, Table 2), leading to the formation of the product complexes **C**_**(*****R*****)**_ and **C**_**(*****S*****)**_. We note that the latter complexes have different relative energies (Figure 5); however, the free (*R*)- and (*S*)-allyl carbamate enantiomers of **3** of course have identical energies, with an overall computed driving force for the conversion of CO_2_, propylamine, and cinnamyl chloride to allyl carbamate of −4.2 kcal/mol.

From the energy profile (Figure 5), we conclude that for the formation of the (*R*)-product, the first step **TS**_**AB(*****re*****)**_ has a higher barrier than **TS**_**BC(*****R*****)**_ by 2.8 kcal/mol, making the former the rate-determining TS (provided that the final step from **C** to **A** is not rate-limiting, which should be a reasonable assumption, given that it is a simple exchange of neutral ligands, Scheme 2).^53^ In contrast, for the formation of the (*S*)-enantiomer of the product, the situation is reversed, with the second step, **TS**_**BC(*****S*****)**_, being rate-limiting, displaying a barrier that is 2.5 kcal/mol higher than the first step, **TS**_**AB(*si*)**_. Thus, the computed energies indicate a significant preference for the (*S*)-pathway, with an overall barrier of 17.6 kcal/mol, compared to the overall barrier of 20.3 kcal/mol for the (*R*)-pathway.^49^ With chloride as a nucleophile, both reaction steps favor the formation of the (*S*)-enantiomer (Tables 1 and 2); thus, this preference appears not to be dependent on the nucleophile.

To evaluate the robustness of the computed results, we reoptimized **TS**_**AB**_ and **TS**_**BC**_ with the ωB97XD and B3LYP-D3 functionals (SI, Table S1). The absolute energies of the transition states vary with the different functionals (see the SI), but the relative energy differences indicate similar selectivities. All three DFT functionals predict **TS**_**AB**_ and **TS**_**BC**_ as the rate-limiting steps for the (*R*)- and (*S*)-pathways, respectively, and favor the formation of the (*S*)-enantiomer. Thus, the mechanistic details and the predicted major product enantiomer agree across different computational protocols.

Intriguingly, the experimentally reported *e.e.* for formation of **3** is 94% (*R*).^20^ We note that this assignment was based on an X-ray structure of the related product ((*R*)-1-(4-bromophenyl)allyl isopropylcarbamate); thus, it may not apply to **3** (for a discussion of the stereochemical assignment, see the SI). Furthermore, in a later study, with the same catalyst and substrates and similar reaction conditions (albeit with the substitution of solvent and base by DMSO and K_3_PO_4_),^28^ the *e.e.* was reported as 35% (*S*) for formation of **3**. Our additional calculations with DMSO as the solvent show results analogous to those with toluene (see the SI, Table S3). Overall, we predict that the (*S*)-enantiomer of **3** is the major species formed.

### Formation of Side Products

The experimental report indicated the formation of two side products, **4** and **5** (Scheme 1).^18^ The linear achiral product **4** is formed through the attack of the propyl carbamate on the terminal carbon (C_t_) of the allyl. The computed transition state **TS**_**BC(t)**_ has a barrier of 19.1 kcal/mol, which is 1.5 kcal/mol higher than that of **TS**_**BC**_ (17.6 kcal/mol, Scheme 2). On the basis of the barriers for attack at the benzylic versus the terminal carbon, the theoretically predicted ratio of products **3:4** is ∼94:6, in good agreement with the experimentally observed ratio 90:10.^18^

The allyl amine **5** was also reported as a side product (Scheme 1). We have tested possibilities for the formation of this intermediate from species **A** and **B**, but the computed barriers are unfeasible (see Scheme S2 in the SI). Therefore, we propose that **5** is formed externally from **3**, without the aid of the iridium complex.

## Conclusions

In this work, we studied the mechanism of the iridium-catalyzed allylic substitution reaction to produce branched allylic carbamates with high enantiopurity. Our computational analysis shows that the benzylic carbon of the allyl fragment prefers to remain *trans* to the phosphorus atom of the phosphoramidite ligand in the most stable isomer **II**. The iridium complex does not activate CO_2_, and instead, the nucleophilic carbamate is formed through a reaction of CO_2_ with the free amine, assisted by DABCO. In the reaction between the carbamate and the iridium-bound allyl, the rate-determining step for the (*R*)- and (*S*)-pathways differs, being, respectively, the oxidative addition of cinnamyl chloride (**TS**_**AB**_) and the nucleophilic attack of propyl carbamate on the allyl (**TS**_**BC**_). Our computed mechanism predicts the (*S*)-enantiomer as the major product with both carbamate and chloride nucleophiles. The results indicate that the nucleophile itself does not play a deterministic role in dictating the enantioselectivity of this reaction. Our insights are vital to understand the enantioselectivity and regioselectivity of the allylic carbamate product, allowing for a more rational approach toward designing new reactions involving the enantioselective synthesis of allylic carbamates with CO_2_.

## Supporting Information Available

Additional computational results (PDF)
XYZ coordinates of the optimized geometries (XYZ)

## Supplementary Material

om4c00177_si_001.pdf

om4c00177_si_002.xyz
