# Supplementary material for: Computational Study of the Ir-Catalyzed Formation of Allyl Carbamates from CO2
Source: Organometallics. 2024 Aug 5;43(17):1818–26. doi: 10.1021/acs.organomet.4c00177 (PMC11388460; doi:10.1021/acs.organomet.4c00177)
Supplement: Supplementary file 1 — om4c00177_si_001.pdf [file om4c00177_si_001.pdf]

Supporting Information

Computational Study of the Ir-catalyzed Formation of Allyl Carbamates from CO_2

Sahil Gahlawat,[a,b] Markus Artelsmair,[c] Abril C. Castro,[d] Per-Ola Norrby,*[,e] Kathrin H. Hopmann*[,a]

[a]Department of Chemistry, UiT The Arctic University of Norway, N-9017 Tromsø, Norway. Contact email: kathrin.hopmann@uit.no
[b]Hylleraas Centre for Quantum Molecular Sciences, UiT The Arctic University of Norway, N-9017 Tromsø, Norway
[c]Isotope Chemistry, Early Chemical Development, Pharmaceutical Sciences, R&D, AstraZeneca Gothenburg, SE-431 83 Mölndal, Sweden
[d]Hylleraas Centre for Quantum Molecular Sciences, Department of Chemistry, University of Oslo, P.O. Box 1033 Blindern, 0315 Oslo, Norway
[e]Data Science and Modelling, Pharmaceutical Sciences, R&D, AstraZeneca Gothenburg, SE-431 83 Mölndal, Sweden. Contact email: Per-Ola.Norrby@astrazeneca.com

Table of Contents

1. Alternative Mechanisms S2
   1.1. Initial amine-coordination S2
   1.2. Formation of 5 .... S4
2. AIMD simulations of B_{re} S5
3. Models of DABCO, CO_2 and propylamine S6
4. Results with other DFT functionals S7
5. Stereochemical assignment S8
6. Results with DMSO solvent S10

7. Non-covalent interactions in $TS_{BC(t)}$ and $TS_{BC(S)}$……………………………………………………..…..…S11

8. References.. S12

1. Alternative mechanisms

1.1 Initial amine-coordination: In addition to the mechanism shown in the main text (Scheme 2), we studied an alternate mechanistic pathway involving coordination of amine to the iridium complex. The computed pathway is shown in Scheme S1.

The Ir(I)-complex **A** is formed through cyclometallation of **L1** and the coordination of cinnamyl chloride as discussed in the main text (Scheme 2a). In the alternative mechanism, the amine replaces cinnamyl chloride to form complex **B1**. In the next step, the carbamate ion is formed by attack of amine on CO_2 with a barrier of 21.3 kcal/mol. Notably, the formation of carbamate occurs via an outer sphere mechanism, i.e. CO_2 does not interact with Ir metal. The optimized structure of the transition state is shown in Figure S1, in which the iridium makes non-covalent interactions with the hydrogen atom of the amine. An inner sphere transition state for the formation of carbamate could not be located.

The formed intermediate **C1** displays an uncoordinated carbamate species. The computed energy of **C1** is higher than that of the CO_2 insertion TS (Scheme S1), which may be due to the occasional underestimation of barriers by DFT.[1–3] Oxidative addition of cinnamyl chloride to **C1** has a computed barrier of 15.1 kcal/mol and provides **B**, an Ir(III) η^3-allyl complex also present in the main mechanism (main text Scheme S2b). In the next step, carbamate performs a nucleophilic attack on the benzylic carbon of the allyl to give the product complex **C**. The subsequent replacement of the carbamate product by cinnamyl chloride concludes the catalytic cycle. Compared to the mechanism presented in the main text (Scheme 2b), the initial complex **B1** is 8.2 kcal/mol higher in energy than complex **B**. This indicates that **B** will be more abundant than **B1**, and the reaction will likely proceed via the mechanism in the main text. Also, the CO_2 insertion energy barrier (21.3 kcal/mol) for the altenative mechanism is higher than the highest barrier in the main mechanism (20.3 kcal/mol, Figure 5).

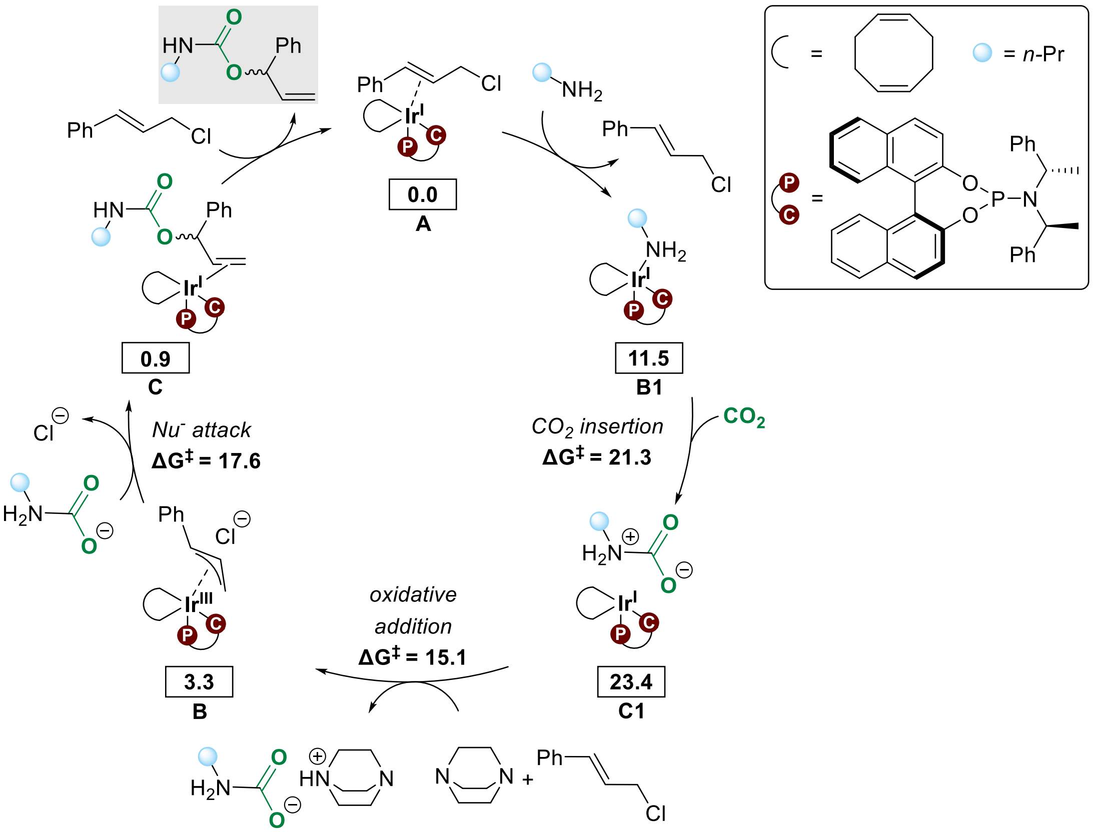

Scheme S1. An alternate mechanism computed for the allylic substitution reaction. Free energies are at 298 K (kcal/mol, PBE0-D3(BJ)/def2TZVPP,SDD[Ir](PCM)//PBE0-D3(BJ)/def2SVP,SDD[Ir](PCM). The energetic reference state is complex **A**.

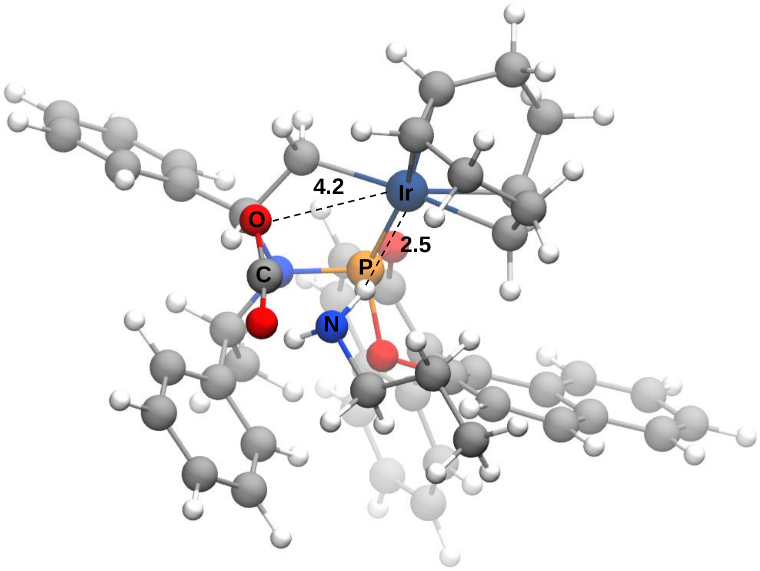

Figure S1. The optimized geometry of the CO_2 insertion transition state in the alternative mechanism (Scheme S1).

1.2. Formation of 5: We investigated the formation of the side product **5** from species **A** and **B** as shown in Scheme S2. The attack of amine on the benzylic carbon of the allyl in **A** has a barrier of 40.4 kcal/mol, which is unfeasible under the given reaction conditions.

The attack of amine on the benzylic carbon of the allyl in **B** provides complex **F2** with a barrier of 20.9 kcal/mol. DABCO subsequently abstracts a proton from **F2** to give product complex **E2**. The barrier from **B** to **C** is 17.6 kcal/mol, which is 3.3 kcal/mol lower than the barrier from **B** to **F2**. This shows that formation of **5** from **B** is not favorable. Therefore, we propose that **5** is formed externally from **3** without the aid of the iridium complex.

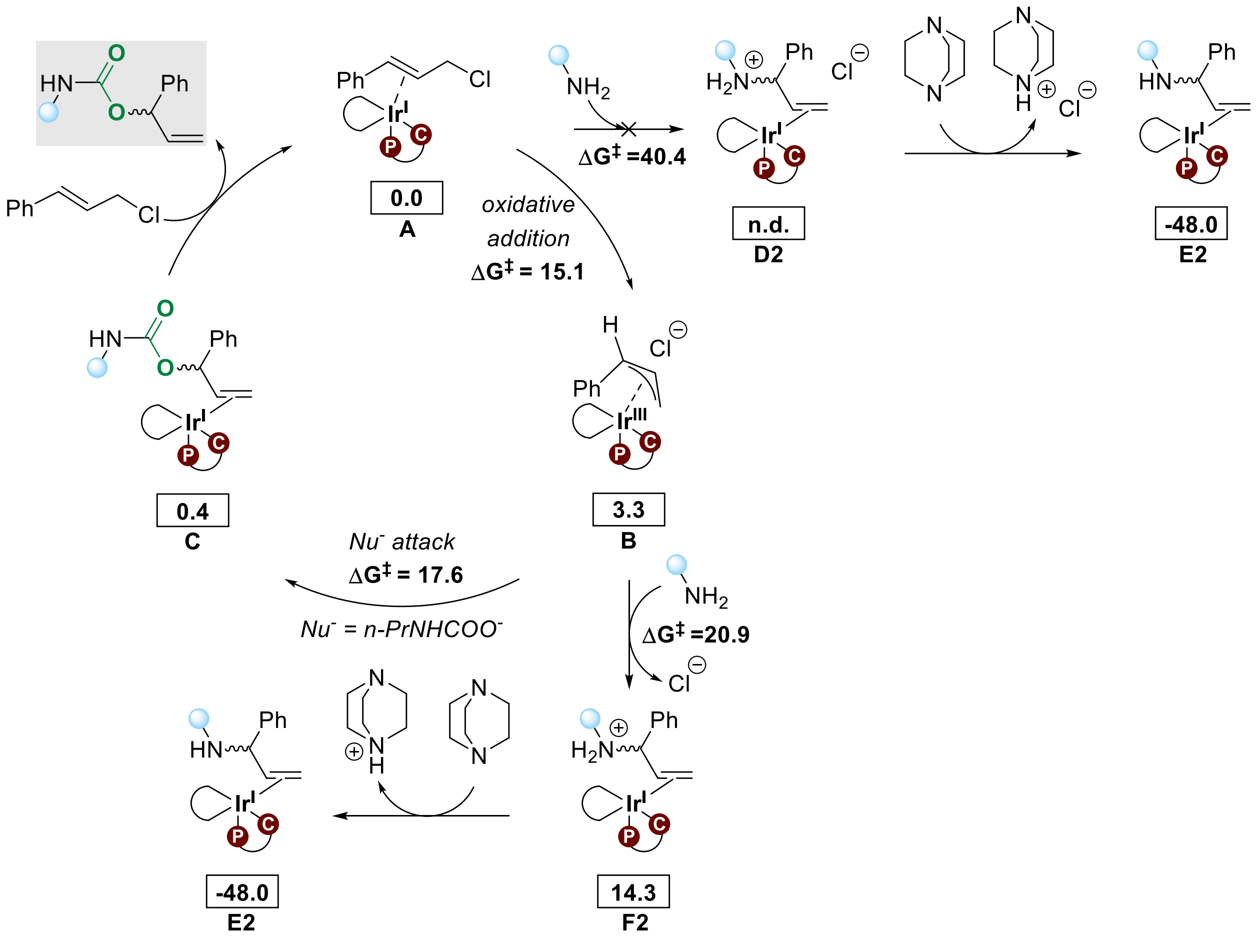

Scheme S2. Formation of side product **5**. Gibbs free energies are at 298 K (kcal/mol, PBE0-D3(BJ)/def2TZVPP,SDD[Ir](PCM)//PBE0-D3(BJ)/def2SVP,SDD[Ir](PCM). The energetic reference state is complex **A**. (n.d. = geometries cannot be optimized)

2. AIMD simulations of B*re*

We performed ab-initio molecular dynamics (AIMD)[4] simulations of intermediate **B*re*** to investigate the behavior of the released counter ion (Cl^-). The calculations were performed with explicit solvent molecules of toluene according to the Born-Oppenheimer approximation using the CP2K program package.[5] The initial model for the AIMD simulations was generated using the Packmol program.[6] The model has 34 explicit solvent molecules of toluene around **B*re*** in a simulation cubic box of edge 20.1 Å, reproducing the solvent density of 0.86 g/mL. The simulation cell was treated under periodic boundary conditions and using a time step of 0.25 fs. The simulation was run with Kohn-Sham DFT using the PBE exchange-correlation functional,[7,8] with a combined DZVP Gaussian[9] and auxiliary plane-wave (250 Ry cutoff) basis set. The core electrons were represented using pseudopotentials of the Goedecker–Teter–Hutter (GTH) type.[10] Dispersion interactions were accounted for with the Grimme's D3 model.[11] The initial system was relaxed using a micro canonical ensemble (NVE) until it reached a target temperature of 298 K. After the equilibration, a production trajectory of 25 ps was generated using a canonical (NVT) ensemble at an average temperature of 298 K regulated with the CSVR algorithm.[12]

The evolution of the Ir−Cl^- bond distances over time is shown in Figure S2. The mean distance found is 4.7 Å. The plot indicates that the chloride ion does not coordinate to the iridium atom during the simulation time.

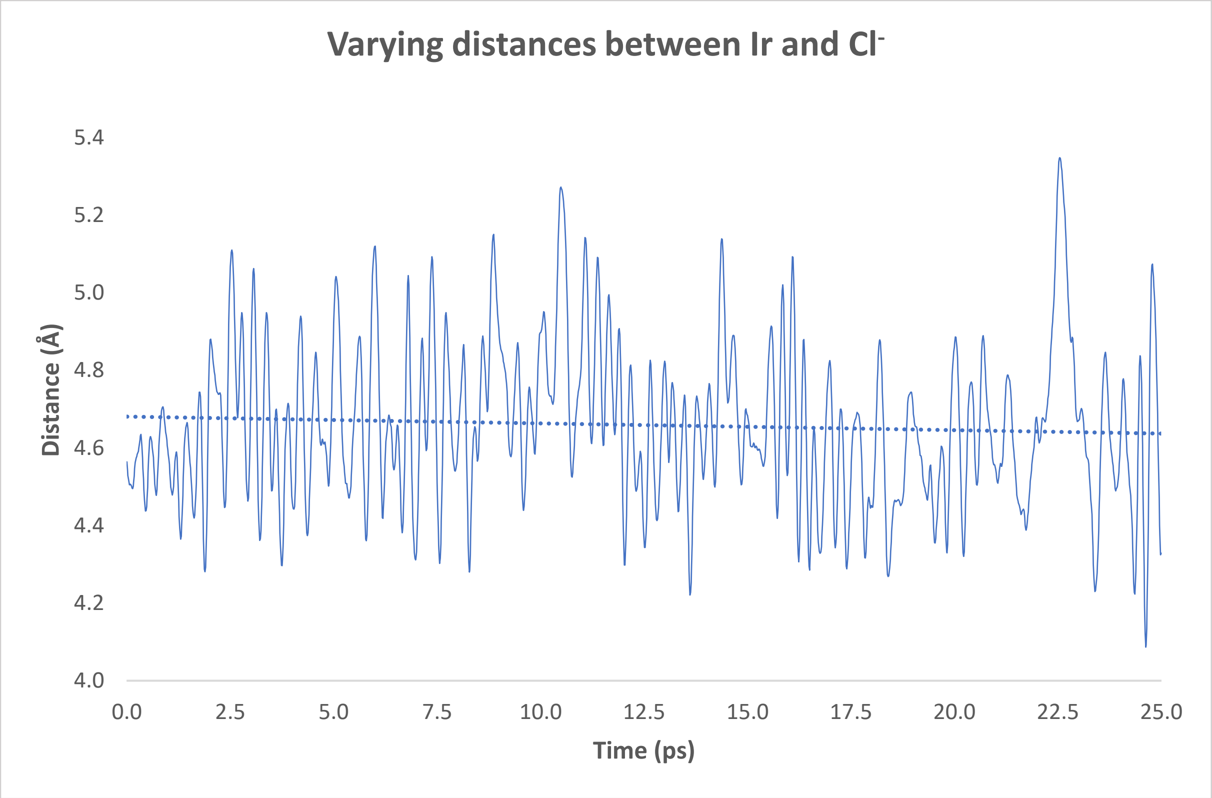

Figure S2. Bond distance between Ir and Cl^- in **B*re*** during 25 ps of the NVT trajectory.

3. Models of DABCO, CO_2 and propylamine

Based on our computations, we predict that propylamine and CO_2 combine to form a carbamate ion nucleophile, with DABCO deprotonating the amine. We tested the different species that can be formed from DABCO, CO_2 and propylamine (Scheme S3). The DABCO-carbamic acid complex **F** has the lowest energy and thus it is used as the species in the catalytic cycle presented in the main text. We were unable to optimize the TS for attack of the amine on CO_2, as during the optimization process, the bond is immediately formed, indicating that the carbamate formation has an insignificant barrier. Species **iv** has a hydrogen bond between amine and DABCO, with CO_2 being modelled separately. The species **v** represents a protonated DABCO and a separately computed carbamate ion formed by attack of amine on CO_2. This species is significantly higher in energy than **F**, due to charge separation. Species **vi** represents the separately computed neutral DABCO and neutral carbamaic acid, i.e. the hydrogen bond interaction present in **F** is missing.

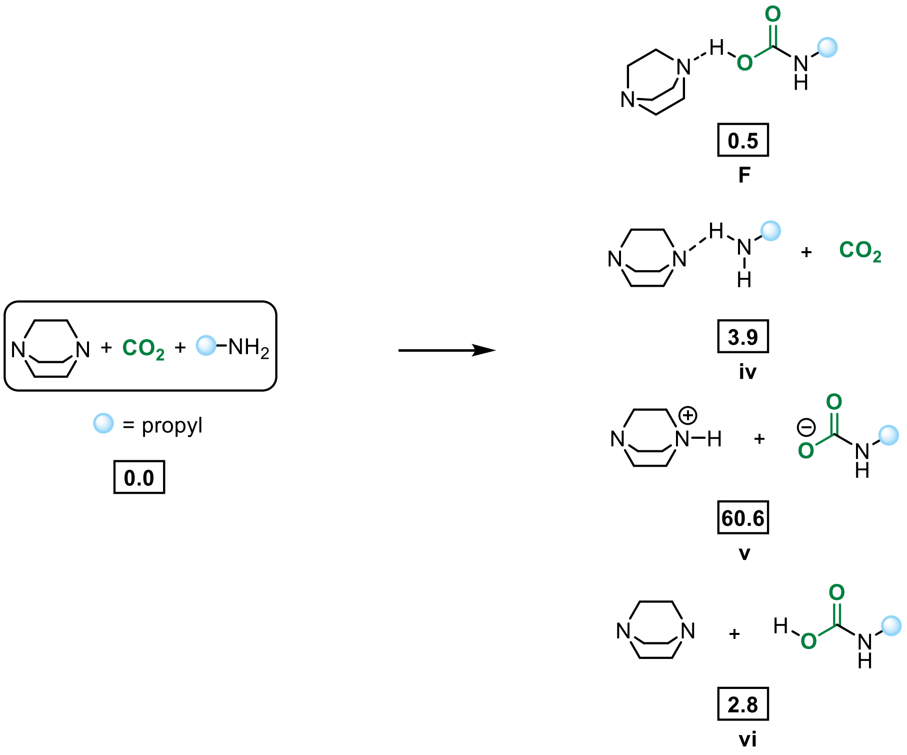

Scheme S3. Different species that can be formed from propylamine, CO_2 and DABCO. Energies are given in kcal/mol relative to the separated molecules (left side).

4. Results with other DFT functionals

We computed the most stable conformation **II** for **TS$_{AB}$** and **TS$_{BC}$** (carbamate (*n*-PrNHCOO$^-$) as nucleophile) with two additional DFT functionals. Table S1 displays the energy barriers for TSs with ωB97XD, dispersion-corrected PBE0-D3(BJ), and B3LYP-D3(BJ) functionals. Notably, the relative energies between TSs remain similar with different computational protocols, indicating the robustness of our results. All the functionals predict the (*S*)-enantiomer to be the major product.

Table S1. Calculated barriers for **TS$_{AB}$** and **TS$_{BC}$** with wb97XD functional.

Transition State	$\Delta G^{\ddagger}_{PBE0\text{-}D3BJ}$ (kcal mol^{-1})	$\Delta G^{\ddagger}_{\omega B97XD}$ (kcal mol^{-1})	$\Delta G^{\ddagger}_{B3LYP\text{-}D3BJ}$ (kcal mol^{-1})
**TS$_{AB}$-II-*re***	20.3	20.5	14.8
**TS$_{AB}$-II-*si***	15.1	16.8	10.2
**TS$_{BC}$-II-*R***	17.5	17.4	12.2
**TS$_{BC}$-II-*S***	17.6	17.5	12.7

5. Stereochemical assignment

Our calculations for cinnamyl chloride show that the predicted enantioselectivity is similar for both carbamate and chloride nucleophiles, suggesting that selectivity of the (*S,S,S*a)-**L1**-iridium-catalyzed allylic substitution does not depend on the nucleophile (Table 1 and 2, main text). Both models predict the preferred formation of the (*S*)-enantiomer. Two experimental studies utilizing the same (*S,S,S*a)-**L1**-iridium catalyst have reported different selectivities for the reaction of cinnamyl chloride, propyl amine and CO_2, with the enantiomeric excess of the resulting allyl carbamate reported as respectively 94 % (*R*) and 35 % (*S*) (see Scheme 4 in the experimental paper).[13,14] For the former study, the absolute configuration of all products was assigned as (*R*) based on the X-ray structure of the allyl carbamate product obtained from 4-bromo-cinnamyl chloride (94 % ee, from hexane, CCDC 970275).[13] In order to evaluate if the configuration may depend on the substrate, we calculated $\mathbf{TS_{AB}}$ and $\mathbf{TS_{BC}}$ with 4-bromo-cinnamyl chloride. The barriers for the conformations **I-VIII** with chloride as nucleophile are shown in Table S2. The results predict that conformation **II** is energetically preferred, as for cinnamyl chloride, with the preferred formation of the (*S*)-enantiomer of the product. Figure S3 shows the corresponding optimized geometries of $\mathbf{TS_{AB}}$ and $\mathbf{TS_{BC}}$ (conformation **II**). Thus, all computationally tested substrates and nucleophiles predict formation of the (*S*)-product with (*S,S,S*a)-**L1**-iridium. We cannot exclude that there exists an alternative (and energetically preferred) (*R*)-pathway that we have not identified in our computational work. Alternatively, it may be possible that the experimental assignment of the major allyl carbamate formed from 4-bromo-cinnamyl chloride as (*R*) may represent a misassignment.[13]

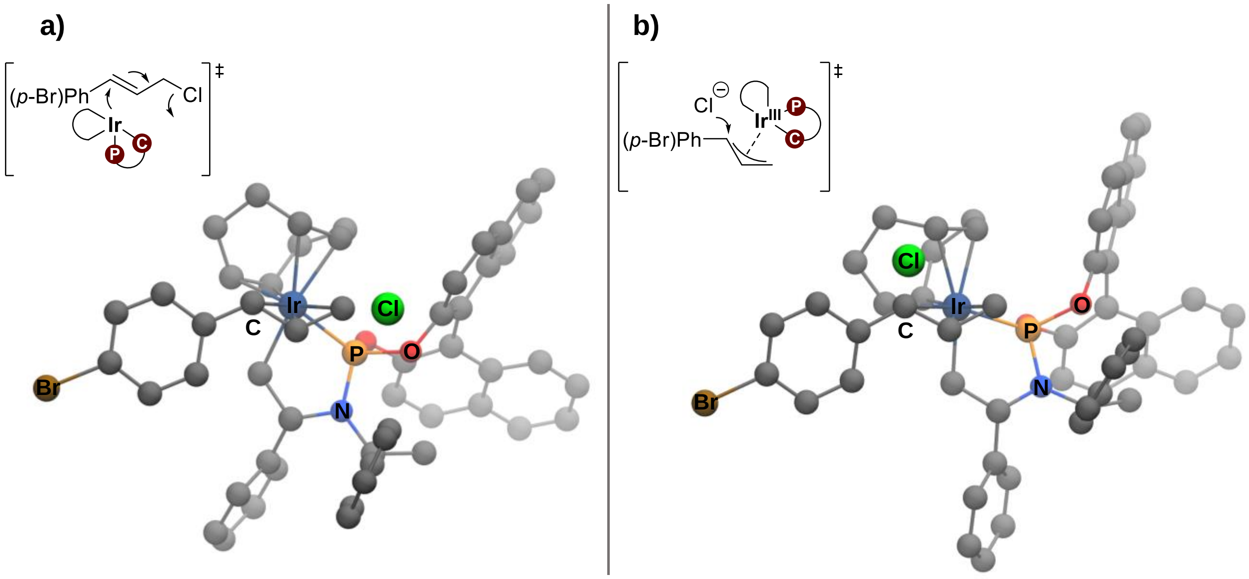

Figure S3. The optimized transition state geometries for **a)** oxidative addition of 4-bromo-cinnamyl chloride and **b)** nucleophilic attack of chloride on the 4-bromo-cinnamyl chloride. Conformation **II** is shown for both TSs.

Table S2. Calculated $\mathbf{TS_{AB}}$ and $\mathbf{TS_{BC}}$ barriers for the 4-bromo-cinnamyl chloride substrate.

$\mathbf{TS_{AB}}$	$\boldsymbol{\Delta G^{\ddagger}_{re}}$	$\boldsymbol{\Delta G^{\ddagger}_{si}}$
TS_{AB}-I-Br	n.d.	20.2
TS_{AB}-II- Br	**20.9**	**15.5**
TS_{AB}-III- Br	22.7	32.4
TS_{AB}-IV- Br	n.d.	n.d.[a]
TS_{AB}-V- Br	28.1	n.d.[a]
TS_{AB}-VI- Br	19.9	n.d.[a]
TS_{AB}-VII- Br	32.2	29.0
TS_{AB}-VIII- Br	26.9	29.2

$\mathbf{TS_{BC}}$	$\boldsymbol{\Delta G^{\ddagger}_{R}}$	$\boldsymbol{\Delta G^{\ddagger}_{S}}$
TS_{BC}-I_{cl}-Br	19.7	20.2
TS_{BC}-II_{cl}-Br	**16.1**	**13.4**
TS_{BC}-III_{cl}-Br	21.9	23.2
TS_{BC}-IV_{cl}-Br	25.9	20.2
TS_{BC}-V_{cl}-Br	20.1	19.5
TS_{BC}-VI_{cl}-Br	19.5	23.2
TS_{BC}-VII_{cl}-Br	25.9	23.1
TS_{BC}-VIII_{cl}-Br	24.3	24.8

[a](n.d. = geometries cannot be optimized)

6. Results with DMSO solvent

We computed the most stable conformation **II** for **TS$_{AB}$** and **TS$_{BC}$** (carbamate (*n*-PrNHCOO$^-$) as nucleophile) with DMSO solvent. Table S3 displays the energy barriers for TSs with toluene and DMSO as the solvents. Notably, the relative energies between TSs remain similar. Both solvents favor the formation of the (*S*)-enantiomer.

Table S3. Calculated barriers for **TS$_{AB}$** and **TS$_{BC}$** in DMSO solvent.

Transition State	$\Delta G^{\ddagger}_{Toluene}$ (kcal mol^{-1})	$\Delta G^{\ddagger}_{DMSO}$ (kcal mol^{-1})
**TS$_{AB}$-II-*re***	20.3	12.2
**TS$_{AB}$-II-*si***	15.1	8.4
**TS$_{BC}$-II-*R***	17.5	8.8
**TS$_{BC}$-II-*S***	17.6	8.4

7. Non-covalent interactions

NCI plots with the non-covalent interactions between carbamate and the iridium complex for **TS**$_{\mathbf{BC(}\boldsymbol{R}\mathbf{)}}$, **TS**$_{\mathbf{BC(}\boldsymbol{S}\mathbf{)}}$, and **TS**$_{\mathbf{BC(t)}}$ are displayed in Figure S4. The nucleophilic oxygen of carbamate makes attractive interactions (in green) with the benzylic carbon of the allyl in all TSs. The repulsive interactions (in red) are formed between the allyl and the carbon atom of carbamate. The propyl chain of the carbamate interacts via van der Waals forces (in green) with the phosphoramidite and allyl ligands. The van der Waals interactions are more pronounced at **TS**$_{\mathbf{BC(t)}}$ where the nucleophile approaches the terminal carbon atom of the allyl. For carbamate attack at the benzylic carbons, the nucleophile exhibits comparable non-covalent interactions at **TS**$_{\mathbf{BC(}\boldsymbol{R}\mathbf{)}}$ and **TS**$_{\mathbf{BC(}\boldsymbol{S}\mathbf{)}}$, which is in line with the similar barriers for these two TSs.

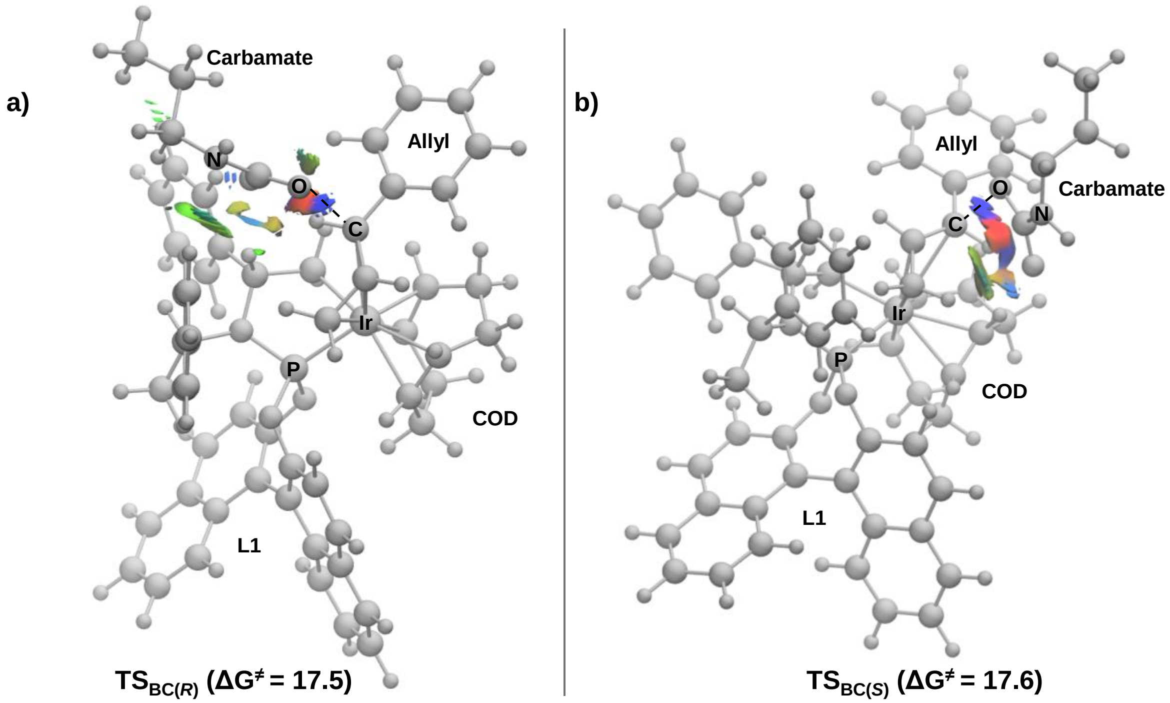

c)

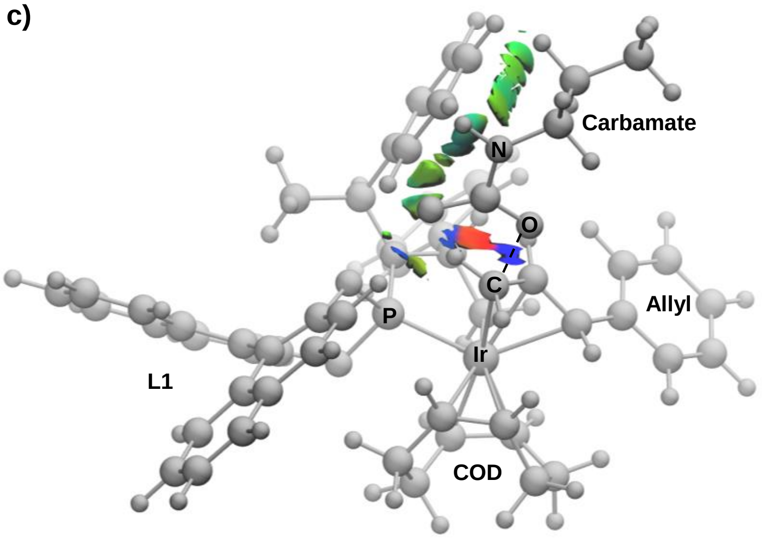

$TS_{BC(t)}$ ($\Delta G^{\neq}$ = 19.1)

Figure S4. Non-covalent interactions at the optimized geometries of **a) $TS_{BC(R)}$**, **b) $TS_{BC(S)}$**, and **c) $TS_{BC(t)}$** (isomer **II**, carbamate as nucleophile). The gradient isosurfaces (s = 0.3 au) are colored on a blue-green-red scale corresponding to values of sign(λ2)ρ, ranging from −3 to 3 au. Blue indicates strong attractive interactions, green represents weak van der Waals interactions, and red indicates strong repulsive interactions. A dashed black line is drawn between the atoms forming bonds and the ligands are labelled. TS barriers computed at 298 K (kcal/mol, PBE0-D3(BJ)/def2-TZVPP,SDD[Ir](PCM)//PBE0-D3(BJ)/def2-SVP,SDD[Ir](PCM).

8. References

(1) Patchkovskii, S.; Ziegler, T. Improving “Difficult” Reaction Barriers with Self-Interaction Corrected Density Functional Theory. *J. Chem. Phys.* **2002**, *116* (18), 7806–7813. https://doi.org/10.1063/1.1468640.

(2) Shukla, P. B.; Mishra, P.; Baruah, T.; Zope, R. R.; Jackson, K. A.; Johnson, J. K. How Do Self-Interaction Errors Associated with Stretched Bonds Affect Barrier Height Predictions? *J. Phys. Chem. A* **2023**, *127* (7), 1750–1759. https://doi.org/10.1021/ACS.JPCA.2C07894.

(3) Bryenton, K. R.; Adeleke, A. A.; Dale, S. G.; Johnson, E. R. Delocalization Error: The Greatest Outstanding Challenge in Density-Functional Theory. *Wiley Interdiscip. Rev. Comput. Mol. Sci.* **2023**, *13* (2). https://doi.org/10.1002/WCMS.1631.

(4) Iftimie, R.; Minary, P.; Tuckerman, M. E. Ab Initio Molecular Dynamics: Concepts, Recent Developments, and Future Trends. *Proc. Natl. Acad. Sci. U. S. A.* **2005**, *102* (19), 6654–6659. https://doi.org/10.1073/PNAS.0500193102.

(5) Kühne, T. D.; Iannuzzi, M.; Del Ben, M.; Rybkin, V. V.; Seewald, P.; Stein, F.; Laino, T.; Khaliullin, R. Z.; Schütt, O.; Schiffmann, F.; Golze, D.; Wilhelm, J.; Chulkov, S.; Bani-Hashemian, M. H.; Weber, V.; Borštnik, U.; Taillefumier, M.; Jakobovits, A. S.; Lazzaro, A.; Pabst, H.; Müller, T.; Schade, R.; Guidon, M.; Andermatt, S.; Holmberg, N.; Schenter, G. K.; Hehn, A.; Bussy, A.; Belleflamme, F.; Tabacchi, G.; Glöß, A.; Lass, M.; Bethune, I.; Mundy, C. J.; Plessl, C.; Watkins, M.; VandeVondele, J.; Krack, M.; Hutter, J. CP2K: An Electronic Structure and Molecular Dynamics Software Package -Quickstep: Efficient and Accurate Electronic Structure Calculations. *J. Chem. Phys.* **2020**, *152* (19), 194103. https://doi.org/10.1063/5.0007045/199081.

(6) Martinez, L.; Andrade, R.; Birgin, E. G.; Martínez, J. M. PACKMOL: A Package for Building Initial Configurations for Molecular Dynamics Simulations. *J. Comput. Chem.* **2009**, *30* (13), 2157–2164. https://doi.org/10.1002/JCC.21224.

(7) Perdew, J. P.; Burke, K.; Ernzerhof, M. Generalized Gradient Approximation Made Simple. *Phys. Rev. Lett.* **1996**, *77* (18), 3865. https://doi.org/10.1103/PhysRevLett.77.3865.

(8) Perdew, J. P.; Burke, K.; Ernzerhof, M. Generalized Gradient Approximation Made Simple [Phys. Rev. Lett. 77, 3865 (1996)]. *Phys. Rev. Lett.* **1997**, *78* (7), 1396. https://doi.org/10.1103/PhysRevLett.78.1396.

(9) Godbout, N.; Salahub', D. R.; Andzelm' And, J.; Wimmer, E. Optimization of Gaussian-Type Basis Sets for Local Spin Density Functional Calculations. Part I. Boron through Neon, Optimization Technique and Validation. *Canadian Journal of Chemistry* **2011**, *70* (2), 560–571. https://doi.org/10.1139/V92-079.

(10) Goedecker, S.; Teter, M. Separable Dual-Space Gaussian Pseudopotentials. *Phys. Rev. B* **1996**, *54* (3), 1703. https://doi.org/10.1103/PhysRevB.54.1703.

(11) Grimme, S.; Antony, J.; Ehrlich, S.; Krieg, H. A Consistent and Accurate Ab Initio Parametrization of Density Functional Dispersion Correction (DFT-D) for the 94 Elements H-Pu. *J. Chem. Phys.* **2010**, *132* (15), 154104. https://doi.org/10.1063/1.3382344.

(12) Bussi, G.; Donadio, D.; Parrinello, M. Canonical Sampling through Velocity Rescaling. *J. Chem. Phys.* **2007**, *126* (1), 14101. https://doi.org/10.1063/1.2408420/186581.

(13) Zhang, M.; Zhao, X.; Zheng, S. Enantioselective Domino Reaction of CO2, Amines and Allyl Chlorides under Iridium Catalysis: Formation of Allyl Carbamates. *Chem. Commun.* **2014**, *50* (34), 4455–4458. https://doi.org/10.1039/c4cc00413b.

(14) Zheng, S. C.; Zhang, M.; Zhao, X. M. Enantioselective Transformation of Allyl Carbonates into Branched Allyl Carbamates by Using Amines and Recycling CO2 under Iridium

Catalysis. *Chem. – A Eur. J.* **2014**, *20* (24), 7216–7221.
https://doi.org/10.1002/CHEM.201402388.
